# Thermosensitive Cationic Magnetic Liposomes for Thermoresponsive Delivery of CPT-11 and SLP2 shRNA in Glioblastoma Treatment

**DOI:** 10.3390/pharmaceutics15041169

**Published:** 2023-04-06

**Authors:** Yu-Jen Lu, Hao-Lung Hsu, Yu-Hsiang Lan, Jyh-Ping Chen

**Affiliations:** 1 Department of Chemical and Materials and Materials Engineering, Chang Gung University, Kwei-San, Taoyuan 33302, Taiwan; 2 Department of Neurosurgery, Chang Gung Memorial Hospital at Linkou, School of Medicine, Chang Gung University, Kwei-San, Taoyuan 33305, Taiwan; 3 Craniofacial Research Center, Chang Gung Memorial Hospital at Linkou, Kwei-San, Taoyuan 33305, Taiwan; 4 Research Center for Food and Cosmetic Safety, College of Human Ecology, Chang Gung University of Science and Technology, Taoyuan 33302, Taiwan; 5 Department of Materials Engineering, Ming Chi University of Technology, Tai-Shan, New Taipei City 24301, Taiwan

**Keywords:** liposomes, chemotherapy, drug delivery, magnetic nanoparticles, cancer therapy

## Abstract

Thermosensitive cationic magnetic liposomes (TCMLs), prepared from dipalmitoylphosphatidylcholine (DPPC), cholesterol, 1,2-distearoyl-sn-glycero-3-phosphoethanolamine-N-[methoxy(polyethylene glycol)]-2000, and didodecyldimethylammonium bromide (DDAB) were used in this study for the controlled release of drug/gene for cancer treatment. After co-entrapping citric-acid-coated magnetic nanoparticles (MNPs) and the chemotherapeutic drug irinotecan (CPT-11) in the core of TCML (TCML@CPT-11), SLP2 shRNA plasmids were complexed with DDAB in the lipid bilayer to prepare TCML@CPT-11/shRNA with a 135.6 ± 2.1 nm diameter. As DPPC has a melting temperature slightly above the physiological temperature, drug release from the liposomes can be triggered by an increase in solution temperature or by magneto-heating induced with an alternating magnetic field (AMF). The MNPs in the liposomes also endow the TCMLs with magnetically targeted drug delivery with guidance by a magnetic field. The successful preparation of drug-loaded liposomes was confirmed by various physical and chemical methods. Enhanced drug release, from 18% to 59%, at pH 7.4 was observed when raising the temperature from 37 to 43 °C, as well as during induction with an AMF. The in vitro cell culture experiments endorse the biocompatibility of TCMLs, whereas TCML@CPT-11 shows some enhancement of cytotoxicity toward U87 human glioblastoma cells when compared with free CPT-11. The U87 cells can be transfected with the SLP2 shRNA plasmids with very high efficiency (~100%), leading to silencing of the SLP2 gene and reducing the migration ability of U87 from 63% to 24% in a wound-healing assay. Finally, an in vivo study, using subcutaneously implanted U87 xenografts in nude mice, demonstrates that the intravenous injection of TCML@CPT11-shRNA, plus magnetic guidance and AMF treatment, can provide a safe and promising therapeutic modality for glioblastoma treatment.

## 1. Introduction

Glioblastoma multiforme (GBM) is one of the most devastating primary brain cancers found in adults. Although improvements in surgical techniques and advancements in therapeutic strategies were made recently, GBM is often associated with an inexorable, rapidly fatal clinical course with a median survival time less than 15 months [1,2]. The key factor responsible for this bleak prognosis is the malignant nature of GBM, where highly infiltrative tumor cells can invade different parts of brain tissues and result in incomplete surgical resection, as well as a high recurrence rate [3]. CPT-11 (Irinotecan), which functions by inhibiting the DNA eukaryotic enzyme topoisomerase I of cancer cells, is one of the chemotherapeutic drugs for treating GBM [4,5]. However, a major hurdle impeding successful chemotherapy is the non-selective nature of the cytotoxic chemotherapeutic agent in differentiating between normal and malignant cells, which usually results in detrimental effects on healthy tissue, but without causing pronounced cytotoxicity toward cancer cells [6]. Therefore, it is important to develop a targeted drug delivery strategy for CPT-11, such as one utilizing magnetic targeting, in order to minimize its adverse side effects.

Encapsulation of pharmaceutical agents in a liposomal carrier provides numerous advantages for drug delivery, including high drug-loading capacity, biocompatibility, biodegradability, and low cytotoxicity [7]. They can also protect the drug from degrading in the physiological environment, offering improved therapeutic efficacy [8]. A stimulus-responsive liposomal system can further enhance therapeutic efficacy by releasing the encapsulated drug specifically within the tumor, when triggered by an external signal, such as temperature change, resulting in more accurate control of drug delivery [9]. By not triggering the drug to release in normal tissues, the unwanted adverse effects could be prevented [10]. Inducing drug release from thermoresponsive liposomes is of particular interest where temperature change in a biological milieu can increase the bioavailability and therapeutic index of the encapsulated drug [11]. Although these liposome-based drug delivery systems offer many promising advantages over conventional methods of chemotherapy, their therapeutic efficacy is still limited [12]. In fact, tumor vascular permeability varies among different tumor types, which can result in unpredictable liposomal accumulation at tumoral tissue via extravasation [13]. Therefore, in order to address these obstacles, a versatile drug delivery system can be designed by encapsulating magnetic nanoparticles (MNPs) into liposomes for the preparation of thermosensitive magnetic liposomes [14]. Using a strong permanent gradient magnetic field, the magnetic liposomes can be guided to a target site for drug delivery in cancer therapy [15]. Indeed, magnetic targeting is a promising drug delivery strategy in which drug carriers can be concentrated locally in the targeted site in order to minimize side effects [16]. On the other hand, as the MNPs can transform the electromagnetic energy into heat when exposed to an alternating magnetic field (AMF) [17], triggered drug release could be expected from thermosensitive magnetic liposomes after induction with an AMF [18]. Another important application of magnetic liposomes is for T2-weighted magnetic resonance imaging (MRI) to non-invasively monitor their distribution, using MNPs as image contrast agents [19].

On the other hand, chemotherapy might not be capable of efficiently eliminating all malignant cells, as drug resistance to a single chemotherapeutic drug may arise, where drug-resistant cells can evade the apoptotic pathways induced by a cytotoxic drug [20]. Additionally, other factors, such as the heterogeneity of malignant tumors and the existence of cancer cells with different growth rates, may also impede the efficacy of chemotherapy [21]. Therefore, combination cancer therapy, combining different kinds of therapeutic agents, may improve treatment outcomes by thwarting drug resistance [22]. Indeed, recent studies have shown that a combination of chemotherapy with gene therapy can lead to enhanced antitumor efficacy through synergistic effects [23]. Considering gene therapy, the RNA interference (RNAi) is a promising gene regulatory approach that uses small interfering RNA (siRNA), which can be an effective treatment modality for GBM by down-regulating essential genes involved in proliferation, migration, and metastasis of malignant brain cancer cells [24]. However, delivery of the negatively charged siRNA to the cytoplasm in order to exert the RNAi function is challenging, and considerable obstacles must be overcome [25]. First, the siRNA is not stable in vivo, could be degraded rapidly by the endonuclease in the serum, so its half-life can be as short as 15 min [26]. Secondly, the cellular uptake of siRNA is quite low, as its negative charge leads to electrostatic repulsion against the cell membrane with negative membrane potential [27]. In order to overcome the barriers of systemic siRNA delivery, nanoparticles and cationic polymers, as well as liposomes, have been developed as siRNA carriers [28]. Among them, the cationic liposome remains one of the most widely studied non-viral vectors for gene delivery, with its excellent biocompatibility and biodegradability [27]. However, a problem associated with the cationic liposomal siRNA delivery system is low transfection efficiency, which may be improved by using a magnetic field to guide the magnetic liposomal siRNA delivery system [29]. Therefore, magnetic cationic liposomes, with MNPs incorporated in the aqueous core of cationic liposomes, were developed in this study with the aim of enhancing the transfection efficiency.

A clear understanding of the molecular mechanisms responsible for the invasiveness of glioma is of great clinical value, as the infiltrative behavior of glioma cells represents a major factor that contributes to the bleak prognosis of GBM. The stomatin-like protein 2 (SLP2), or the STOML2, is a major protein on the mitochondrial inner membrane. The SLP2 was reported to be overexpressed in many human cancer tissues (including gliomas), and plays an important role in tumor progression and occurrence [30,31]. More importantly, the up-regulation of SLP2 is correlated with the overall survival time of glioma patients. and with the World Health Organization (WHO) histological grade of gliomas, in that patients showing a higher level of SLP2 expression usually also show a shorter survival time [32]. By suppressing the expression of SLP2 by inhibiting the nuclear factor κB/matrix metalloproteinase-9 (NF-κB/MMP9) pathway, the migration ability of glioma cells can be reduced substantially [33]. Furthermore, by depleting SLP2 expression, the sensitivity of cancer cells to chemotherapy-induced apoptosis could be enhanced [33]. Taken together, considering that SLP2 plays an important role in the progression of glioma, silencing of the SLP2 gene via siRNA may be a promising strategy to improve the therapeutic efficacy of glioma [34]. Considering drug/gene delivery by liposomes for cancer therapy, the co-delivery of sorafenib and VEGF-siRNA by pH-sensitive liposomes was used for synergistic hepatocellular carcinoma therapy [35]. Similarly, a thermosensitive magnetic cationic liposomal system was used for the co-delivery of doxorubicin and SATB1 shRNA for the treatment of gastric cancer [36].

In this study, we aim to develop a multifunctional liposomal drug/gene delivery system for GBM treatment by combining magnetic targeting and thermally triggered drug release. The resulting nanocomposite was prepared by entrapping citric-acid-coated Fe_3_O_4_ MNP (CMNPs) and CPT-11 in thermosensitive cationic magnetic liposomes (TCMLs), followed by complexing with SLP2 shRNA plasmids (TCML@CPT-11/shRNA). The formulation can be targeted to the tumor site using magnetic targeting, wherein the triggered release of therapeutic agents could be realized by AMF-induced hyperthermia. The physico-chemical properties of TCML@CPT-11/shRNA, as well as the drug release characteristics, were characterized throughout the preparation step. In order to determine the in vitro anticancer efficacy, the human glioblastoma cell line U87 was used to investigate cell cytotoxicity, cell apoptosis, gene transfection, and gene silencing. Finally, the in vivo antitumor efficacy was studied in U87 tumor-bearing xenografts in nude mice.

## 2. Materials and Methods

### 2.1. Materials

The phospholipid dipalmitoylphosphatidylcholine (DPPC), 1,2-distearoyl-sn-glycero-3-phosphoethanolamine-N-[methoxy(polyethylene glycol)-2000] ammonium salt (DSPE-PEG2000) and 1,2-distearoyl-sn-glycero-3-phosphoethanolamine-N-[amino(polyethylene glycol)-2000] ammonium salt (DSPE-PEG2000-NH_2_) were purchased from Avanti Polar Lipids Inc. (Alabaster, AL, USA). Didodecyldimethylammonium bromide (DDAB), cholesterol, and 5(6)-carboxyfluorescein N-hydroxysuccinimide ester (NHS-fluorescein) were procured from Sigma-Aldrich (St. Louis, MO, USA). The cell culture reagents, fetal bovine serum (FBS), and Dulbecco’s Modified Eagle’s Medium (DMEM) were obtained from Life Technologies (Carlsbad, CA, USA). The dyes for confocal microscopy, LysoTracker Red DND-99 (LysoTracker), and diamidino-2-phenylindole (DAPI) were acquired from Thermo Fisher Scientific (Waltham, MA, USA).

### 2.2. Synthesis of Citric-Acid-Coated Magnetic Nanoparticle (CMNP)

The citric-acid-coated magnetic nanoparticle (CMNP) was prepared by following a chemical co-precipitation method [14]. In brief, FeCl_3_·6H_2_O (4.75 g) and FeCl_2_·4H_2_O (1.75 g) were dissolved in 80 mL distilled deionized water (ddH_2_O) in a three-neck flask. The solution was purged for 60 min with N_2_ to prevent oxidation and heated to 60 °C. Next, 10 mL NH_4_OH (28%) was slowly added to maintain the solution pH at 10, and stirred at 1000 rpm for 30 min. For citric acid coating, citric acid (0.5 g/mL) was added drop-wisely to the mixture and reacted at 95 °C for 90 min. Dialysis tubing with a 30 kDa molecular weight cutoff was used for dialysis against ddH_2_O, and the purified CMNP solution was stored at 20 mg/mL concentration in ddH_2_O at 4 °C.

### 2.3. Preparation of Thermosensitive Cationic Magnetic Liposomes (TCMLs)

The thin-film hydration method was used to prepare CMNP-encapsulated thermosensitive cationic magnetic liposomes (TCMLs). A 1 mL chloroform solution containing 90 mol% DPPC, 5 mol% cholesterol, 5 mol% DDAB, and 0.5 mol% DSPE-PEG2000 was prepared in a round-bottomed flask. After evaporating the chloroform with a rotary evaporator (EYELA, Tokyo, Japan) at 100 psi and 50 °C for 30 min, a thin lipid film was formed around the wall of the flask. The lipid film was further dried overnight in a vacuum chamber, followed by rehydration of the lipid film with a CMNP (20 mg/mL)/CuSO_4_ (0.3 M) solution prepared in phosphate buffer (pH 7.4, 10 mM) for 20 min at 50 °C. The hydrated solution was sonicated at 150 W power for 10 min and extruded through a 200 nm polycarbonate membrane in a commercial temperature-controlled barrel extruder (Lipex extruder, Transferra Nanosciences, Burnaby, BC, Canada). In order to encapsulate CPT-11 in TCMLs (TCML@CPT-11), 1 mL TCML solution, prepared as above; 10 μL of calcimycin (0.2 mM,); and 0.1 mL CPT-11 solution were mixed by stirring at 150 rpm and 50 °C for 20 min. The solution went through five freezing-thawing cycles in liquid nitrogen with 1 min freezing time and 10 min thawing time, followed by centrifugation for 30 min at 65,000× *g* to remove impurities. In order to visualize the intracellular uptake of TCMLs, fluorescein-labeled liposomes were prepared by replacing DSPE-PEG2000 with DSPE-PEG2000-NH_2_ during the preparation of the TCML solution. The NHS-fluorescein was mixed with the TCMLs in a 2:1 ratio at room temperature for 1 h, to conjugate its NHS groups spontaneously with the amine groups on the surface of the TCMLs. After adding 1 M glycine to block unreacted amine groups, the fluorescein-labeled TCML solution was obtained via centrifugation for 30 min at 30,000× *g*.

### 2.4. Physico-Chemical Characterization

The zeta potential, particle size, and polydispersity (PDI) of CMNPs and liposomes were obtained via dynamic light scattering (DLS) using a Nano ZA90 Zetasizer (Malvern, Worcestershire, UK) at 25 °C. The sample was diluted with ddH_2_O for DLS measurements. The Fourier transform infrared (FTIR) spectra were recorded using a Horiba FT-730 FTIR spectrometer (Horiba, Tokyo, Japan), after blending the sample with KBr powder. The sample was scanned at 2.5 mm/s from 400 to 4000 cm^−1^ with 4 cm^−1^ resolution. The morphology of the liposomes was observed via transmission electron microscopy (TEM) using a cryo-TEM (JEOL JEM-1400, Tokyo, Japan) at 120 kV and −170 °C. The sample was prepared by dropping a liposome solution onto a 200-mesh carbon-coated copper grid and fixed by freezing in liquid ethane using Cryoplunger 3. The fixed specimen was mounted into a Gatan cryoholder for cryo-TEM observation. The suspension stability of the CMNP solution and liposomes at 37 °C in pH 7.4 phosphate-buffered saline (PBS) was assessed from gross observation and by measuring the solution absorbance at 410 nm (OD_410_) for up to 7 days.

For differential scanning calorimetry (DSC) analysis, the sample prepared in ddH_2_O (10 mg) was placed in a DSC aluminum pan and analyzed using a Q20 differential scanning calorimeter (TA Instruments, New Castle, DE, USA) under nitrogen at a 50 mL/min gas flow rate. The temperature increase rate was 5 °C/min from 20 °C to 60 °C. For X-ray diffraction (XRD) analysis, a D2 Phaser X-ray powder diffractometer (Bruker, Madison, WI, USA) was used to scan dried power from 25° to 80° (2θ) with 0.02°/s step size using Cu Kα radiation. The phase was compared with the JCPDS database for identification, and the crystalline size was calculated using the Debye–Scherrer equation. Thermogravimetric analysis (TGA) was performed using a Q50 thermogravimetric analyzer (TA Instruments, New Castle, DE, USA). A powder sample (8 mg) was heated in nitrogen from 10 to 650 °C at a 10 °C/min heating rate. The magnetization property was determined using a superconducting quantum interference device (SQUID) magnetometer (MPMS XL-7, Quantum Design, San Diego, CA, USA) at 25 °C from −10,000 to 10,000 Gauss. The weight percentage of Fe_3_O_4_ MNP entrapped in liposomes was determined via inductively coupled plasma optical emission spectrometry (ICP-OES, Varian 710-ES, Santa Clara, CA, USA).

### 2.5. Drug Loading and Release

The amount of CPT-11 encapsulated in TCML@CPT-11 was directly measured after breaking the liposomes with Triton X-100. After centrifugation, the concentration of CPT-11 in the supernatant was determined via high performance liquid chromatography (HPLC) using a JASCO LC-4000 HPLC system. A 250 mm × 4.6 mm Eclipse XDB C18 reversed-phase column was used and the mobile phase was 40% (*v*/*v*) 0.01 M phosphate buffer (pH 4) and 60% (*v*/*v*) acetonitrile, while the wavelength for detection was 370 nm [14]. The drug encapsulation efficiency (%) was defined as follows: weight of CPT-11 encapsulated ÷ weight of TCMP@CPT-11 × 100. The drug-loading efficiency (%) was defined as follows: weight of CPT-11 encapsulated ÷ weight of CPT-11 initially added × 100.

For drug release, in vitro temperature-dependent and pH-dependent CPT-11 release from TCML@CPT-11 (5 mg/mL) were investigated at 37 °C and 43 °C in 1 mL PBS (pH 5.4 or pH 7.4) in the dark. The solution was shaken at 120 rpm for drug release and centrifuged for 30 min at 65,000× *g* at predetermined times. The supernatant was totally removed and replenished with 1 mL PBS of the same pH value as previously used, in order to continue drug release. The cumulative amount of CPT-11 released from TCML@CPT11 was quantified using HPLC and calculated as follows: cumulative weight of CPT-11 released ÷ initial weight of CPT-11 in TCML@CPT-11 × 100 [37]. As the CMNPs in the TCML solution could significantly elevate the temperature of liposomes in the presence of a high-frequency AMF to trigger drug release, the AMF-induced drug release was also tested. An Eppendorf tube containing 1 mL TCML@CPT11 (5 mg/mL) in PBS (pH 7.4) was exposed to an AMF at 96 kHz and 60 A with a 3.2 cm-inner diameter coil. The cumulative drug release was calculated using the same formula as above.

### 2.6. In Vitro Cell Culture

#### 2.6.1. Cell Line and Cell Culture Condition

The human primary glioblastoma cell line U87, obtained from the American Type Culture Collection (ATCC HTB1, Manassas, VA, USA), was used in the study. The cell line was modified genetically with lentiviral infection and was able to stably express firefly luciferase in order to facilitate in vivo bioluminescence imaging. The cell culture medium was DMEM supplemented with 10% FBS and 1% penicillin/streptomycin, and the cell culture condition was set to 37 °C in a humidified 5% CO_2_ incubator.

#### 2.6.2. Intracellular Uptake

For the intracellular uptake of liposomes, 1 × 10^5^ U87 cells were seeded onto a 15 mm glass slide in a 24-well cell culture plate and cultured overnight. The fluorescein-labeled TCMLs (green fluorescence) were added to each well, and the culture plate was incubated for another 12 h. The treated cells were washed three times with PBS and fixed for 30 min with 4% paraformaldehyde. The lysosomes were stained for 60 min with LysoTracker (red fluorescence), and the nuclei were stained for 10 min with DAPI (blue fluorescence). The intracellular uptake efficiency of the liposomes was determined from the green fluorescence signal associated with TCMLs. The excitation and emission wavelength for confocal laser scanning microscopy (BioRad Radiance MRP2100, Hercules, CA, USA) were 577/492/340 nm (red/green/blue) and 590/517/488 nm (red/green/blue), respectively.

#### 2.6.3. Transfection of U87 Cells and Cell Migration Assay

A SLP2-shRNA-PGLV3 plasmid or a SLP2-shRNA-PGLV3/GFP plasmid that was able to co-express green fluorescent protein (GFP) and SLP2 shRNA was constructed for the purpose of analyzing the SLP2 gene expression. Both plasmids contained a TOOLSilent pGLV3/H1/GFP/Puro vector, as well as a 19-nucleotide-long shRNA sequence for gene silencing of SLP2 [34].

The TCML/shRNA complexes were prepared by mixing 1 μg of TCMLs with the SLP2-shRNA-PGLV3 in different weight ratios from 20 to 200. The complexes were run on 0.8% agarose gel in Tris-acetate-EDTA Buffer for 25 min at 100 V. The electrophoretic mobility of the samples was investigated by quantifying the extent of shRNA complexation with the TCMLs at different TCML/shRNA weight ratios. Naked plasmids or empty liposomes were used as controls.

In order to investigate the efficiency of SLP2 shRNA transfection, U87 cells were transfected with SLP2-shRNA-PGLV3/GFP. After 5 × 10^3^ cell/well U87 cells were seeded into a 24-well cell culture plate and cultured for 24 h, a TCML/shRNA sample with varying TCML/shRNA ratios, from 20 to 200 (*w/w*), was added to each well and the cells were cultured for another 3 days. After removing the medium from each well and washing with PBS, the transfection efficiency was measured from the percentage of cells expressing GFP green fluorescence by observing the samples with an inverted fluorescence microscope (Olympus IX-71, Tokyo, Japan).

The TCML/shRNA prepared with TCML:shRNA = 100 was used to transfect U87 cells and to test the migration of transfected cells using the wound-healing assay [38]. Cells treated with TCMLs alone were used as a control. A 2-well silicone insert (2 Well in µ-Dish 35 mm) was placed in a cell culture dish. The U87 cells were treated with TCMN/shRNA for 2 days and any non-transfected cells were removed by treating them with puromycin. The cells were seeded into the insert at 2 × 10^4^ cell density and cultured to confluency. By removing the silicone strip, a cell-free zone was created at the center of the cell layer, into which cell migration can be observed. In order to observe cell migration as in wound healing, a cell culture was carried out with 1 mL cell culture medium, and images were taken at different times using an optical microscope to monitor the extent of wound closure. The migration ability of U87 cells was quantified by analyzing the area occupied by the migrated cells in the image using the ImageJ software. The recovery of wound area (%) was calculated as: (initial wound area − final wound area) ÷ initial wound area × 100.

#### 2.6.4. In Vitro Cytotoxicity

In order to determine the cytotoxicity of tested samples, U87 cells were seeded into a 96-well cell culture plate (5 × 10^3^ cell/well) and cultured in a humidified 5% CO_2_ incubator at 37 °C for 24 h. After rinsing with PBS (pH 7.4), the cells were incubated with 100 µL of CPT-11 or TCML@CPT-11 solution prepared in culture media containing different amounts of CPT-11, and cultured at 37 or 43 °C for another 24 h. The relative cell viability (normalized to pure cell culture medium) was obtained from the 3-(4,5-dimethylthiazol-2-yl)-2,5-diphenyltetrazolium bromide (MTS) assay, and the solution absorbance was measured at 570 nm (OD_570_) with a microplate reader. The biocompatibility of TCMLs was determined similarly from 0.001 to 500 µg/mL liposome concentration. In order to study temperature-dependent cytotoxicity, 2 × 10^5^ U87 cells were seeded into T-25 cell culture flasks and cultured for 24 h in a humidified 5% CO_2_ incubator. After washing with PBS, the cells were co-cultured with CPT-11 or TCML@CPT11 at 37 °C or 43 °C for 24 h. Following the same protocol, the cytotoxicity was also determined at 37 °C or 43 °C at a drug dosage of 4 μM CPT-11. For flow cytometry analysis of the apoptotic mechanism, 2 × 10^5^ U87 cells were treated with different samples (drug dosage = 150 μM CPT-11) for 4 h. After removing cells from the dish surface with trypsin/EDTA (0.1%), the detached cells were collected by centrifugation and reacted with a FITC-Annexin V/propidium iodide (PI) solution in order to determine the percentage of live cells and dead cells due to apoptosis/necrosis. The flow cytometry analysis was conducted using a CytoFLEX flow cytometer (Beckman Coulter, Brea, CA, USA).

### 2.7. In Vivo Study

#### 2.7.1. Xenograft Tumor Model

The 5-week-old male BALB/c nude mice weighing 15 to 20 g that were used in this study were purchased from the National Laboratory Animal Center (Taipei, Taiwan). All animal experiment protocols followed the procedures established and approved by the Chang Gung University’s Institutional Animal Care and Use Committee (IACUC approval number: CGU109-188, approval date: 4 June 2021). A U87 xenograft tumor model was established by injecting 1 × 10^6^ U87 cells in 200 μL cell culture medium into the right flank of each mouse subcutaneously. The bioluminescence imaging (BLI) intensity of implanted tumors was monitored every day, and when the average BLI intensity reached ∼1 × 10^7^, the animals were used to conduct the experiments.

#### 2.7.2. In Vivo Antitumor Efficacy

In order to evaluate the antitumor efficacy of TCML@CPT11/shRNA under magnetic guidance and AMF treatment, the tumor-bearing mice were randomized into five groups (*n* = 5 in each group) and subjected to intravenous (IV) injection of 200 µL of different samples via the tail vein on days 7, 11, 14, and 18. The study groups include the following: 1, PBS (control); 2, CPT-11 + shRNA (7.5 mg/kg CPT-11 and 2.5 mg/kg shRNA); 3, TCMLs; 4, TCML@CPT-11/shRNA (7.5 mg/kg CPT-11 and 2.5 mg/kg shRNA); and 5, TCML@CPT-11/shRNA (7.5 mg/kg CPT-11 and 2.5 mg/kg shRNA) + AMF treatment (20 mT for 5 min). In each group, magnetic guidance was created by placing a magnet (2400 Gauss) around the implanted tumor site for 30 min [39]. After administration, the tumor size and body weight were recorded continuously for four weeks. The length and width of each tumor were measured using a caliper for calculation of tumor size, as tumor length × (tumor width)^2^ ÷  2. For bioluminescence imaging (BLI), the mice were anesthetized with 1% isoflurane and 100 µL of D-luciferin solution, injected intraperitoneally at 150 mg luciferin/kg body weight dose. A non-invasive in vivo imaging system (IVIS) (Xenogen IVIS-200, Caliper Life Sciences, Hopkinton, MA, USA) was used for BLI in determining the peak bioluminescence from imaging. The BLI intensity was determined from the total bioluminescent signal intensity revealed by the tumor. In order to standardize the BLI intensity, the total BLI signal intensity was normalized with the total signal intensity on day 7, when the treatment started.

#### 2.7.3. Histological and Hematologic Analysis

Tumors were harvested 22 days after the start of treatment and fixed immediately with 10% buffered formalin. After being embedded in paraffin, the samples were sectioned to 3–5 µm thickness and stained with hematoxylin and eosin (H&E). Blood samples were withdrawn from treated mice in each group and compared with those collected from mice in the control group by hematologic analysis.

### 2.8. Statistical Analysis

All data were presented as the mean ± standard deviation (SD). For comparison of significance between groups, one-way analysis of variance (ANOVA) analysis was carried out using the SPSS software. Differences were considered to be significant at *p* < 0.05 level.

## 3. Results and Discussion

### 3.1. Preparation of Liposomes

**Figure 1:**
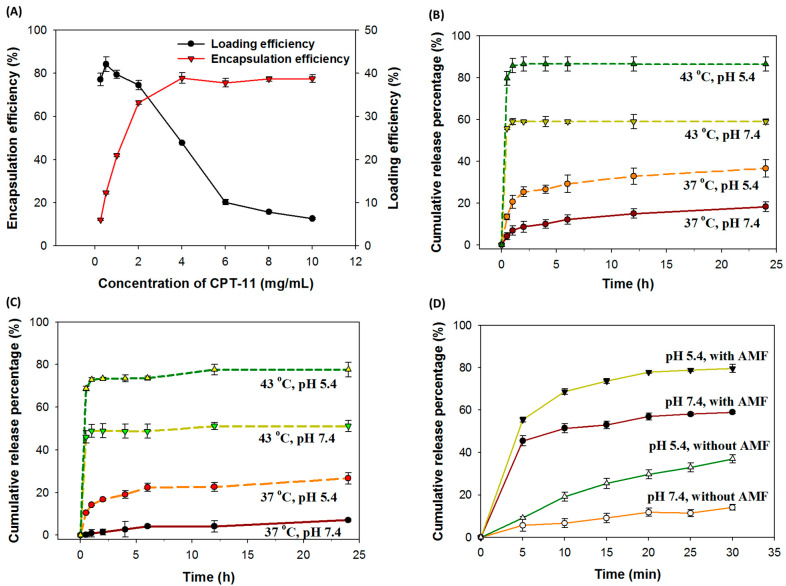
(**A**) The CPT-11 encapsulation efficiency and loading efficiency when a CPT-11 solution with different initial drug concentrations were used for preparation of TCML@CPT-11. (**B**) The pH-sensitive and temperature-sensitive release profiles of CPT-11 from TCML@CPT-11 (5 mg/mL TCML@CPT-11, *n* = 3). (**C**) The pH-sensitive and temperature-sensitive release profiles of CPT-11 from TCML@CPT-11/shRNA (5 mg/mL TCML@CPT-11/shRNA, *n* = 3). (**D**) The pH-sensitive release profiles of CPT-11 from TCML@CPT-11/shRNA when an alternating magnetic field (AMF) (12 mT AMF, 5 mg/mL TCML@CPT-11/shRNA, *n* = 3) is used to increase the temperature.

Since the formulation of drug-loaded liposomes is pivotal in drug development, we started by finding the optimal formulation of TCML@CPT11. The encapsulation efficiency and loading efficiency of CPT-11 in TCML@CPT11 were reported in Figure 1A. When TCML@CPT-11 was prepared by increasing the initial concentration of CPT-11, the drug encapsulation efficiency, which is the weight percentage of CPT-11 in TCML@CPT-11, increased from 6% to 38%. However, the loading efficiency, which is the weight percentage of CPT11 loaded in TCML@CPT-11, decreased from 74% to 12%. Thus, a dramatic increase in the encapsulation efficiency is found to be concomitant with a substantial decrease in the loading efficiency. As a suitable drug formulation should consider both encapsulation efficiency and loading efficiency, we chose 2 mg/mL CPT-11 as the condition for preparation of TCML@CPT-11, which can provide 33% encapsulation efficiency and 74% loading efficiency.

As the release of a loaded drug from drug carriers is desirable after endocytosis for maximal therapeutic efficacy, a pH-responsive or thermoresponsive drug release behavior for TCML-CPT11 should be substantiated. Thermosensitive liposomes, including TCMLs, possess a unique characteristic, in that they show a gel-to-liquid phase transition at a melting phase transition temperature (Tm). Owing to heat generation induced by an AMF to increase the temperature above the Tm of TCML, the structure of the lipid bilayer will be changed, which allows for the release of encapsulated drugs. As shown in Figure 1B for drug release at pH 7.4, 60% CPT-11 was released from TCML@CPT-11 at 43 °C (above the Tm of DPPC) within 1 h, whereas only 8% CPT-11 was released at 37 °C (below the Tm of DPPC), confirming the thermoresponsive drug release behavior of TCML@CPT-11. For pH-sensitive drug release, the experiment was conducted at pH 5.4 in order to simulate the endosomal environment in contrast to the physiological condition (pH 7.4). The percentages of CPT-11 released within 24 h at 37 °C are 36% and 18% at pH 5.4 and 7.4, respectively, and they are 86% and 59% at 43 °C, corroborating the pH-sensitive release behavior of CPT-11 from TCML-CPT-11 (Figure 1B). Overall, only a limited amount of drug (18%) can be released at the physiological conditions (37 °C, pH 7.4) during circulation. In contrast, an increase of up to five-fold in drug release percentage is expected in the endosomal environment (pH 5.4) after intracellular uptake by tumor cells and temperature elevation induced by an AMF for the triggered release of CPT-11. These thermosensitive and pH-sensitive drug release behaviors were also preserved for TCML@CPT11/SLP2shRNA (Figure 1C).

As the presence of CMNPs in TCMLs is expected to elevate the temperature of TCMLs under a high-frequency AMF, further experiments were performed in order to simulate the drug release induced by AMF treatment, where magneto-thermal effects are expected to trigger drug release [40]. As shown in Figure 1D, the amount of CPT-11 released from TCL@CPT11 is pH-dependent as well. Most importantly, an AMF can substantially increase the release of encapsulated CPT-11, where AMF-induced hyperthermia can change the lipid bilayer structure and trigger CPT-11 release. Undoubtedly, the increase in drug release from 14% (pH 7.4 and without AMF) to 80% (pH 5.4 and with AMF) endorses the use of TCML@CPT-11/shRNA for AMF-induced intracellular drug delivery.

**Figure 2:**
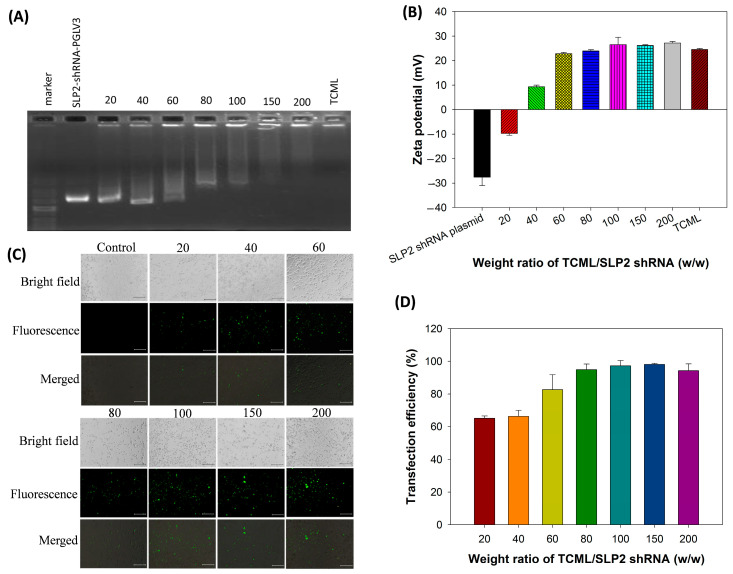
The agarose gel electrophoresis images (**A**), and the zeta potential (**B**) of TCML/shRNA. The TCMLs are bound with SLP2-shRNA-PGLV3 plasmids under different TCML/SLP2 shRNA weight ratios. The fluorescence microscopic images (**C**, bar = 200 μm) and the transfection efficiency (**D**) by transfection of U87 cells with TCML/shRNA. The TCMLs are complexed with TCML/SLP2-shRNA-PGLV3/pGFP plasmids under different TCML/SLP2 shRNA weight ratios. The transfection efficiency was determined from the percentage of transfected U87 cells in each optical microscopy image, which produce green fluorescence protein (GFP) and emit green fluorescence.

A non-viral vehicle for siRNA delivery commonly shows a cationic net charge in order to facilitate the complexation with the negatively charged nucleic acids [41]. Hence, the cationic TCML system was chosen as the vehicle for the delivery of SLP2 shRNA in this study. In order to evaluate the shRNA binding affinity to TCMLs, agarose gel electrophoresis was utilized, by taking advantage of the retention of formed complexes in the wells while only the free shRNA can migrate towards the positive electrode. As shown in Figure 2A, a gradual rise in the retarded proportion of shRNA is found by increasing the TCML/shRNA weight ratio. The shRNA was completely retarded when the weight ratio exceeded 100. Considering the binding of positively charged complexes to the negatively charged cell membrane to promote endocytosis, the zeta potential of TCML/shRNA prepared with different TCML/SLP2 shRNA weight ratios was examined by determining the surface net charge of the vehicle after complexation of the anionic shRNA with the cationic liposomes. As shown in Figure 2B, the zeta potential value of TCML/shRNA changes from negative to positive when the TCML/shRNA ratio reaches 40. The transfection efficiency was determined by identifying U87 cells expressing green fluorescent protein (GFP), which was encoded together with SLP2 shRNA in the plasmid. After being transfected with liposomes prepared with different TCML/shRNA weight ratios for 3 days, the U87 cells showed a different population of GFP-positive cells when examined under a fluorescence microscope (Figure 2C). In order to quantify the transfection efficiency, the percentage of U87 cells transfected with the plasmid was calculated by counting the percentage of U87 cells which were both producing GFP and showing green fluorescence. As illustrated in Figure 2D, TCML/shRNA exhibited an increasing trend of transfection efficiency, from ~60% to ~100% when the ratio of TCML/shRNA increased from 20 to 100. Taken together, the TCML/shRNA prepared with a ratio of TCML/shRNA = 100 was chosen as the preferred condition in which to prepare TCML@CPT-11/shRNA, owing to its efficient shRNA complexation, cationic nature, and excellent transfection efficiency.

### 3.2. Characterization of Liposomes

**Figure 3:**
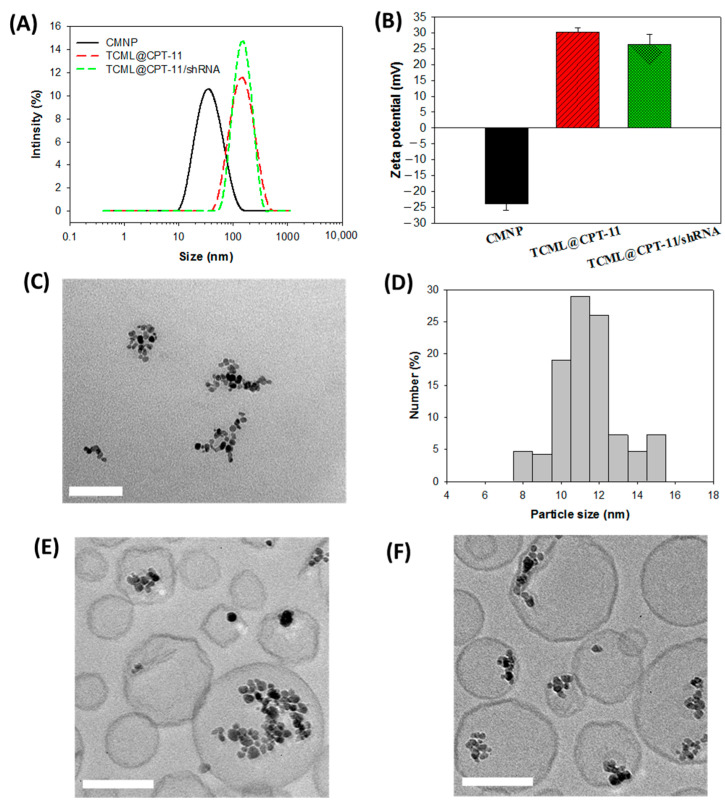
The particle size distribution from dynamic light scattering (**A**), and the zeta potentials (**B**, *n* = 3) of CMNP, TCML@CPT-11, and TCML-CPT-11/shRNA. The TEM image of CMNP (**C**) (bar = 100 nm), and the particle size distribution obtained by counting the size of discrete nanoparticles in the agglomerate (**D**). The cryo-TEM images of TCML@CPT-11 (**E**) and TCML@CPT-11/shRNA (**F**) (bar = 100 nm).

The liposomes were prepared and characterized after each synthesis step. In a nanoparticle system, physico-chemical attributes, such as particle size and polydispersity index (PDI) value, play a crucial role in endocytosis-dependent cellular uptake [42]. From Figure 3A, the dynamic light scattering (DLS) analysis revealed a normal distribution of the hydrodynamic diameters of CMNPs, TCML@CPT-11, and TCML@CPT-11/shRNA, with their average values being 30.2 ± 2.28 nm, 131.7 ± 17.9 nm, and 135.6 ± 2.1 nm, respectively, which can facilitate endocytosis by cancer cells [43]. In a desirable liposome-based drug delivery system, the optimal PDI values are expected to be below 0.3, indicating uniform particle size distribution and good suspension stability [42,44]. The PDI values were 0.212 ± 0.015, 0.128 ± 0.013, and 0.171 ± 0.012 for CMNPs, TML@CPT-11, and TCML-CPT-11/shRNA, respectively. The CMNPs exhibited a highly negative zeta potential value (−23.9 mV) due to the presence of negatively charged carboxylate groups in citrate molecules coated on the particle surface (Figure 3B). The zeta potential changed to 30.2 mV and 26.5 mV for TCML@CPT-11 and TCML@CPT-11/shRNA, respectively, indicating the successful synthesis of TCML-CPT-11/shRNA with the presence of cationic lipid DDAB in the lipid bilayer, and the successful complexation with the negative charged plasmid DNA [45]. TEM analysis of the CMNPs revealed agglomeration of discrete nanoparticles of ~12 nm particle size (Figure 3C,D). The structures of TCML-CPT11 and TCML-CPT-11/shRNA, as characterized from cryo-TEM, revealed successful encapsulation of CMNP in the liposomes, where dark nanoparticles were found in the aqueous core encompassed by a lipid bilayer (Figure 3E,F).

**Figure 4:**
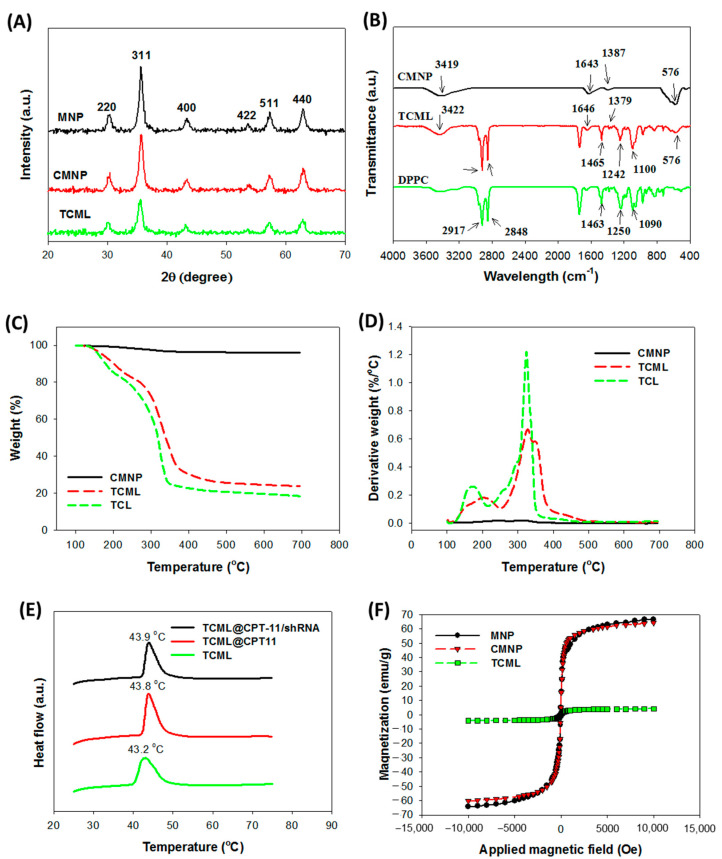
The X-ray diffraction (XRD) analysis (**A**), Fourier transform infrared (FTIR) spectra (**B**), thermogravimetric analysis (TGA) (**C**), differential thermal analysis (DTA) (**D**), differential scanning calorimetry (DSC) thermograms (**E**), and superconducting quantum interference device (SQUID) analysis (**F**) of different samples. MNP—iron oxide magnetic nanoparticles; CMNP—citric-acid-coated iron oxide magnetic nanoparticles; TCL—thermosensitive cationic liposomes; TCML—thermosensitive cationic magnetic liposomes; DPPC—dipalmitoylphosphatidylcholine.

The X-ray diffraction (XRD) patterns of MNPs, CMNPs, and TCMLs are presented in Figure 4A. The powder diffraction patterns show major peaks at 2θ angles 30.5°, 35.9°, 43.5°, 53.5°, 57.0°, and 62.5°, and represent reflection crystal planes of (220), (311), (400), (422), (511), and (440) of a cubic cell, respectively. The position and intensity of the diffraction peaks indicate that the crystalline structure of the particles is identical to that of magnetite from the JCPDS database (card number 19–0629), and supports the presence of pure Fe_3_O_4_ in all samples. Furthermore, the calculated mean crystalline sizes from the strongest diffraction peak (2θ = 35.9°), using the Debye-Scherrer equation, were 11.5 nm, 10.2 nm, and 9.8 nm for MNPs, CMNPs, and TCMLs, respectively, which is consistent with the results observed using TEM.

The Fourier transformed infrared (FTIR) analysis in Figure 4B further confirms the successful preparation of TCMLs. For CMNPs, the presence of Fe_3_O_4_ MNPs could be identified by the major absorption bands at 576 cm^−1^ and 3419 cm^−1^, corresponding to the Fe–O bond in the nanoparticles and the –OH vibrations, respectively. The characteristic bands of citric acid at 1394 cm^−1^ and 1735 cm^−1^, owing to the C–O symmetric vibration and C=O asymmetric stretching from the—COOH group, were shifted to 1387 cm^−1^ and 1643 cm^−1^ in CMNPs. This finding provides direct evidence affirming that citric-acid-coated MNPs were obtained through the chemisorption of carboxylate groups. In addition, the TCMLs demonstrated the characteristic Fe-O peak of CMNPs at 576 cm^−1^, as well as the characteristic peaks of DPPC, the major component of the liposomes, at 1090 cm^−1^ v_s_(PO_2_), 1250 cm^−1^ v_as_(PO_2_), 1463 cm^−1^ (CH_2_), 2848 cm^−1^ v_s_(CH_2_), and 2917 cm^−1^ v_as_(CH_2_). This observation suggests that CMNPs were successfully encapsulated by the DPPC-containing lipid bilayer to form TCMLs.

Thermogravimetric analysis (TGA) (Figure 4C) and differential thermal analysis (DTA) (Figure 4D) were used to analyze the thermal properties and determine the weight percentage of CMNPs loaded in the liposomes. For CMNPs, weight loss starts from ~200 °C and reaches a final residual weight of ~97% (*w*/*w*) at 700 °C, due to the decomposition of surface-coated citric acid [46]. The liposomes prepared without CMNPs (TCLs) show significant weight loss from 110 to 360 °C, and reveal a decomposition peak temperature at ~320 °C, and ~19% (*w*/*w*) residual weight at 700 °C. In comparison with TCLs, TCMLs exhibit similar decomposition behavior, but with better stability and a higher residual weight (24%), as well as a more distinctive peak decomposition temperature at 330 °C, which is caused by the interactions between lipids and CMNPs [47]. The weight percentage of CMNPs in TCMLs could be estimated to be 5.5% from the difference in remaining weight between TCLs and TCMLs at 700 °C, assuming negligible weight loss from CMNPs. This value compares favorably with that obtained from the ICP-OES analysis, which was 6.7 ± 0.1%.

The phase transition behavior of TCML-based liposomes was analyzed by differential scanning calorimetry (DSC) (Figure 4E). As nanoparticles coated with polyethylene glycol (PEG) can overcome the mononuclear phagocyte system and increase their half-lives during circulation, PEGylated TCMLs prepared with 0.5% DSPE-PEG2000 were used in this study [48]. In theory, the DSC calorimetric profiles of DPPC should reveal two phase transition peaks, while only one was observed in TCML-based liposomes, which may be caused by the influence of cholesterol in the liposomes [14]. The endothermic peak temperature is 43.2 °C for TCMLs, which is similar to those of TCML@CPT11 (43.8 °C) and TCML@CPT11/shRNA (43.9 °C), confirming that TCMLs can preserve their thermosensitive characteristics after encapsulating CPT-11 and complexing with shRNA (Figure 4E). From analysis with a superconducting quantum interference device (SQUID) within −10,000 to 10,000 Gauss, minimum remnant magnetization was found for all tested samples (Figure 4F). The saturation magnetization value was 66.7 emu/g for MNPs, 64.2 emu/g for CMNPs, and 4.1 emu/g for TCMLs. The significantly diminished magnetization strength of TCMLs may be a result of the diamagnetic feature of the lipids surrounding the magnetite, together with the low weight percentage of MNPs in TCMLs [49]. No hysteresis loop was found, and the remnant magnetization value of MNPs (0.23 emu/g), CMNPs (0.21 emu/g), and TCMLs (0.27 emu/g) were all close to zero. Taken together, the TCMLs preserve the characteristic superparamagnetic property of MNPs. This property is critical in magnetically targeted cancer therapy after guidance to the target site, since magnetic drug carriers can be easily dispersed by removing the magnetic field in order to avoid agglomeration of nanoparticles and possible vessel blockage [50].

**Figure 5:**
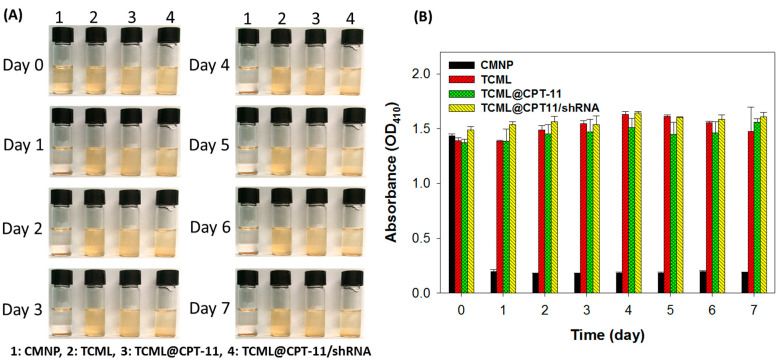
The suspension stability of CMNPs, TCMLs, TCML-CPT11, and TCML-CPT11-shRNA in PBS (pH 7.4) at 37 °C from gross view (**A**), and from the solution absorbance measured at 410 nm (OD_410_) (**B**).

The stabilities of CMNP, TCML, TCML@CPT-11, and TCML@CPT11/shRNA were examined by suspending samples in pH 7.4 PBS at 37 °C. As shown in Figure 5A, CMNPs suffered from precipitation within 1 day, with significantly reduced solution absorbance (OD_410_) compared to other groups (Figure 5B). This indicates agglomeration of CMNPs in PBS. In contrast, the tested liposome samples form well-dispersed solutions with minimum visible precipitation (Figure 5A), as well as with negligible change in solution absorbance (Figure 5B) for up to 7 days, indicating the possibility for intravenous administration.

### 3.3. In Vitro Studies

**Figure 6:**
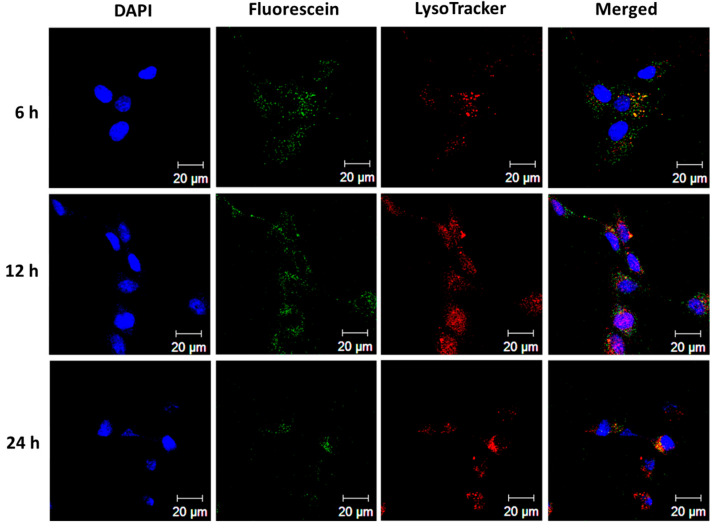
The intracellular uptake of fluorescein-labeled TCMLs (green) by U87 cancer cells after incubating 50 μg/mL TCMLs with the cells for different times. The lysosomes and cell nuclei were stained with LysoTracker (red) and DAPI (blue), respectively, for observation with a laser scanning confocal microscope (bar = 20 µm).

Cationic liposomes are expected to facilitate particle internalization by U87 cells via the interaction between the negatively charged cell membrane and the positively charged liposomes. This passive targeting effect can lead to significant intracellular accumulation of a chemotherapeutic drug, in comparison with administration of a free form of the drug [51]. The intracellular uptake efficiency of green fluorescence-producing TCMLs (labeled with fluorescein) were investigated under a confocal microscope after staining the endosome/lysosome with red fluorescence-producing LysoTracker, and the nucleus with DAPI (blue fluorescence) (Figure 6). After incubating fluorescein-labeled TCMLs with U87 cells for 6 h, the green fluorescence signals corresponding to TCMLs were exclusively co-localized with the red fluorescent signal of lysosomes to show yellow fluorescent spots, suggesting the uptake of TCMLs by U87 cells via endocytosis. Increased green and red fluorescent signals were noted at 12 h, indicating the efficient uptake of TCMLs through charge-mediated endocytosis. As the metabolic enzymes of lysosomes can degrade the internalized liposomes, along with the associated DNA plasmids, nanocarriers for siRNA should escape from the lysosomes after internalization in order to protect the siRNA from enzymatic degradation and, thus, low siRNA delivery efficiency [27]. As shown in Figure 6, the lower localization of TCMLs in the lysosomes at 24 h in comparison with 12 h suggests lysosomal escape of the liposomes. As cationic liposomes can undergo electrostatic-interaction-mediated membrane fusion with the anionic endosome membrane, they can escape from the endosome and release their cargo into the cytoplasm afterwards [52]. Overall, the confocal microscopic analysis indicates that the intercellular uptake of liposomes by U87 cells occurs through an endocytosis internalization pathway.

**Figure 7:**
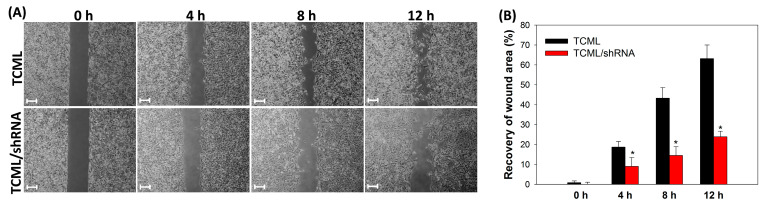
(**A**) The transfection efficiency of TCMLs and TCML/shRNA by comparison of the cell migration ability of transfected U87 cells (bar = 200 μm). (**B**) The quantitative analysis of wound area recovery by normalization of the recovered wound area with the initial wound area. * *p* < 0.05 compared with TCMLs.

The migration ability of invasive glioma cells can be drastically reduced after silencing the SLP2 gene, which also enhances their drug sensitivity to chemotherapy [33]. Therefore, in order to confirm efficient transfection of U87 cells with TCML/shRNA, a wound-healing assay was carried out to elucidate whether the silencing of SLP2 can inhibit the migration ability of U87 cells. As shown in Figure 7A, the migration of U87 cells was inhibited by transfection with TCML/shRNA, in contrast to a control experiment using TCMLs. By calculating the recovered wound area relative to the initial wound area, the values were significantly reduced in the TCML/shRNA group from the TCML group at all time points, presumably due to suppression of the SLP2 gene (Figure 7B). Indeed, the U87 cells transfected with liposomes alone (TCMLs) showed rapid migration and recovered 63% of the created wound within 12 h, in contrast to only 24% when TCML/shRNA was used. Overall, the results support the use of TCMLs as shRNA carriers for SLP2 gene silencing to inhibit the migration of U87 cells, implicating its efficacy for treating invasive U87 tumors in vivo. The expression levels of SLP2 after the in vitro culture were also determined by Western blot and are presented in Figure S1 (Supplementary Materials), which supports effective SLP2 gene silencing in vitro.

**Figure 8:**
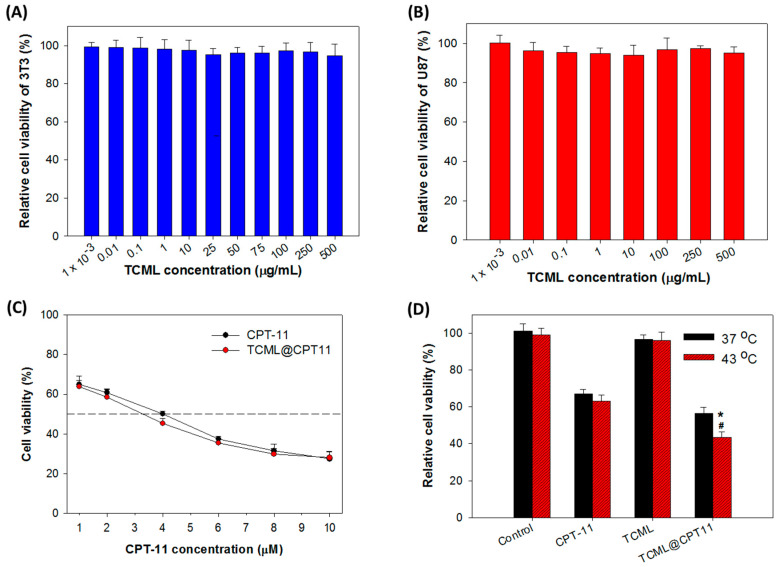
(**A**) The biocompatibility of TCMLs after incubating 3T3 fibroblasts with TCMLs for 24 h (*n* = 6). (**B**) The biocompatibility of TCMLs after incubating U87 cells with TCMLs for 24 h (*n* = 6). (**C**) The cytotoxicity of CPT-11 and TCML@CPT-11 toward U87 cells when tested at different drug concentrations (*n* = 6). (**D**) The temperature-dependent cytotoxicity of different samples towards U87 cells (*n* = 6). The drug concentration is 4 μM CPT-11. The control is cell culture medium. * *p* < 0.05 compared with CPT-11 at 43 °C, ^#^ *p* < 0.05 compared with TCML@CPT11 at 37 °C.

Biocompatibility tests were carried out in order to evaluate the safety of the liposomes. The cell viability test was performed on 3T3 (normal) cells and U87 (cancer) cells, by incubating the cells with different concentrations of TCMLs for 24 h. As shown in Figure 8A, the TCMLs are biocompatible with 3T3 up to 500 μg/mL concentration, as the calculated relative cell viability, in comparison with the cell culture medium, is above 95%. Similar results were also found when incubating TCMLs (10^−3^ to 500 μg/mL) with U87 cells for 24 h, with the relative cell viability still above 95% (Figure 8B).

After substantiating the successful entry of TCMLs into the cells, the cell cytotoxicity of TCML@CPT-11 towards U87 was examined at the same drug concentration as in the free drug. As shown in Figure 8C, TCML@CPT11 exerted a slightly higher cytotoxic effect with lower IC_50_ (3.34 µM), compared with that of CPT-11 (3.85 µM). Although only a partial release of loaded CPT-11 from TCML@CPT11 was noted in Figure 1, efficient intracellular uptake of TCMLs (Figure 6) may up-regulate the cytotoxic effect of TCML@CPT11 toward U87 cells. In order to further investigate the impact of temperature, the cytotoxic effects of CPT-11, TCMLs, and TCML-CPT11 were measured below (37 °C) and above (43 °C) the Tm of DPPC. As illustrated in Figure 8D, no significant differences in cell viability between TCMLs and the control were found at 37 °C and 43 °C, endorsing the biocompatibility of TCMLs. Most importantly, the cell viability of CPT-11 (~65%) was similar at 37 °C and 43 °C, while the cell viability of TCML@CPT11 decreased, from 56% at 37 °C to 40% at 43 °C. Undoubtedly, this is due to the thermoresponsive release of CPT-11 from TCML@CPT11, which can elicit higher cytotoxicity toward cancer cells.

**Figure 9:**
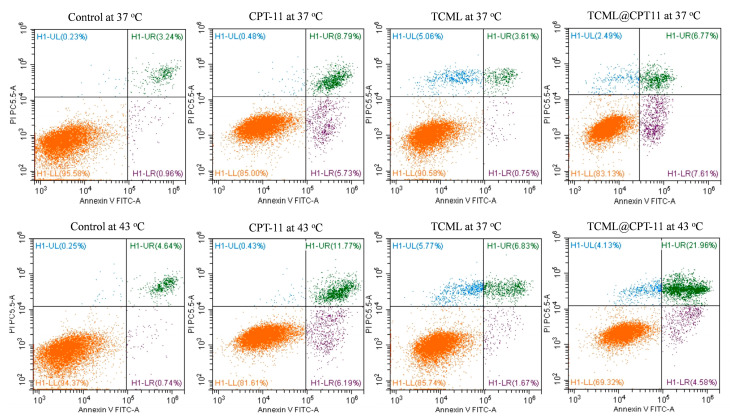
The temperature-dependent apoptosis and necrosis rate of U87 from flow cytometer analysis after staining with Annexin V-FITC/PI. The cells were incubated with different samples containing 150 μM CPT-11 at 37 °C or 43 °C for 4 h. The control is cell culture medium.

The cell apoptosis rate induced by TCML@CPT11 was studied from flow cytometry analysis after staining with Annexin V/propidium iodide (PI). The distribution of live, apoptotic, and necrotic cells was quantified based on the differences in cell membrane permeability between live and apoptotic cells (Figure 9). The flow cytometry data substantiate that the cell death mechanism from CPT-11 occurs mainly through early and late apoptosis. Consistent with the result acquired from the MTS assay (Figure 8), the TCMLs demonstrate high biocompatibility with U87 cells, irrespective of temperature. The cell apoptosis rate only increased from 14.5% to 18.5% for CPT-11 when the temperature was increased from 37 to 43 °C. In contrast, when CPT-11 was encapsulated in thermosensitive liposomes, the apoptotic rate of TCML@CPT-11 increased from 14.4% (37 °C) to 26.5% (43 °C) and revealed the lowest percentage of live cells among all groups. This confirms the thermoresponsive drug release behavior of TCML@CPT-11 as demonstrated from previous results. Taken together, we can confirm that the cytotoxic effect of TCML@CPT11 against U87 cells could be enhanced at a higher temperature, due to triggered drug release from the liposomes. This hyperthermic effect could be facilely accomplished by electromagnetic heating of TCMLs after induction with an AMF.

### 3.4. In Vivo Studies

**Figure 10:**
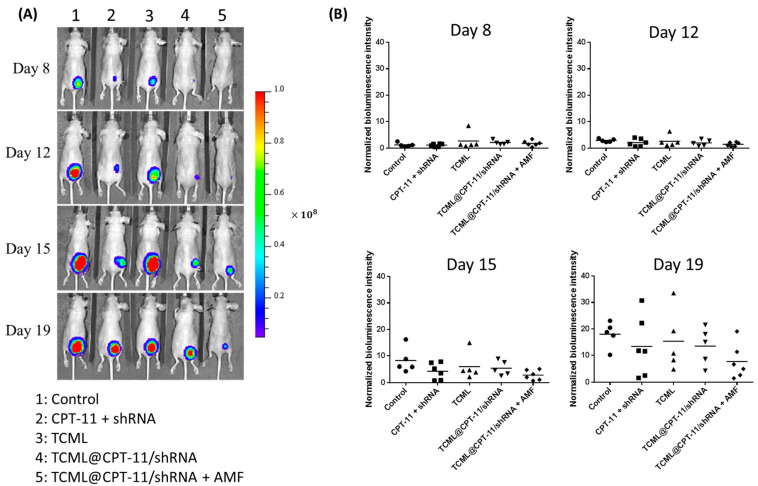
Bioluminescence imaging (BLI) from an in vivo imaging system (IVIS) (**A**), and the normalized bioluminescence intensity (**B**) of U87 tumor-bearing nude mice treated with normal saline (control), CPT-11 + shRNA, TCML, TCML@CPT-11/shRNA, and TCML@CPT11/shRNA + AMF. The dosages of CPT-11 and shRNA were fixed at 7.5 and 2.5 mg/kg body weight, respectively.

The efficacy of TCML@CPT11/shRNA for treating U87 xenografts in nude mice was studied under magnetic guidance and AMF treatment. The U87 tumor-bearing BALB/c mice with U87 cells implanted in the right flank area were divided into five groups (*n* = 5 in each group) when their tumor sizes reached 60–100 mm^3^, and subjected to treatment with PBS (control) or different formulations. Bioluminescence imaging (BLI), using an in vivo imaging system (IVIS), was employed for assessing the treatment efficacy. The IVIS images of tumor-bearing nude mice in each group at different time points are presented in Figure 10A, which clearly shows the best treatment efficacy from the TCML@CPT-11/shRNA + AMF group. The BLI intensity was standardized to the baseline BLI intensity when treatment started (day 7) for each group (Figure 10B). The treatment groups exhibited increasing antitumor treatment efficacy in the order of control < TCML < CPT-11 + shRNA < TCML@CPT-11/shRNA < TCML@CPT-11/shRNA + AMF. Indeed, the TCML@CPT-11/shRNA + AMF group showed significantly lower BLI signal intensity compared to other groups. This enhanced antitumor efficacy can be credited to the triggered release of CPT-11 from TCML@CPT-11/shRNA, which was provided by the magneto-thermal effect induced by an AMF after magnetic targeting, as well as by SLP2 gene silencing with SLP2 shRNA. There was no significant difference between the BLI values of TCML@CPT11/shRNA and CPT-11 + shRNA groups, which is consistent with the previous in vitro results.

**Figure 11:**
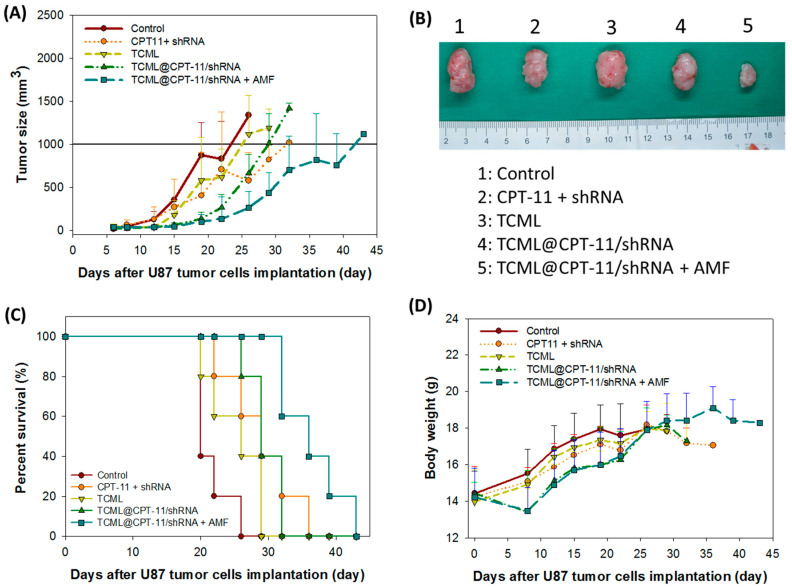
(**A**) The change in tumor size of U87 tumor-bearing nude mice treated with normal saline (control), CPT-11 + shRNA, TCML@CPT-11, TCML@CPT-11/shRNA, and TCML@CPT11/shRNA + AMF. (**B**) The gross view of retrieved tumors on day 22. (**C**) The animal survival curve constructed from the percentage of remaining mice when a tumor-bearing mouse with a tumor volume exceeding 1000 mm^3^ was sacrificed. (**D**) The body weight change with time. The dosages of CPT-11 and shRNA were fixed at 7.5 and 2.5 mg/kg body weight, respectively.

Aside from IVIS, the tumor size after different treatments was continuously monitored throughout the experiments in order to investigate the in vivo antitumor efficacy. As shown in Figure 11A, a rapid increase in tumor volume was noted in the control and TCML groups during the observation period, indicating that TCMLs alone cannot elicit an antitumor effect. Also, the CPT-11 + shRNA and TCML@CPT11/shRNA groups showed only limited suppression of tumor growth. In contrast, the TCML@CPT11/shRNA + AMF group displayed enhanced antitumor efficacy compared with other groups, which was also observed in the BLI analysis. The gross examination of tumor masses explanted on day 22 also provides direct evidence of the antitumor effects of each treatment (Figure 11B). By sacrificing the tumor-bearing mice when the tumor size reached 1000 mm^3^ in each group, a survival curve was constructed from the percentage of remaining mice (Figure 11C). As shown in Table 1, the median survival times of the control and TCML groups were 20 days and 22 days, respectively. The median survival time increased to 29 days for both the CPT11 + shRNA and TCML@CPT-11/shRNA groups, due to the cytotoxic effect of CPT-11, and both groups showed significantly longer average survival times (mean ± SD) than the control. The median survival time dramatically increased to 36 days for treatment with TCML@CPT11/shRNA + AMF, which also provides the longest survival time, significantly higher than all other groups (Table 1). In general, the antitumor efficacy provided from survival time analysis is also consistent with those from the IVIS and tumor size analyses.

In order to evaluate possible adverse effects from each treatment, the body weight of mice was continuously monitored. As shown in Figure 11D, an initial weight loss was noted on day 8 in the TCML@CPT-11/shRNA + AMF group in comparison with the control group, which can be ascribed to the first treatment on day 7. Nonetheless, no significant body weight change was noted following further treatments on days 11, 14, and 18. This suggests minimum systemic toxicity related to the IV administration of TCML@CPT-11/shRNA and the AMF treatment.

**Figure 12:**
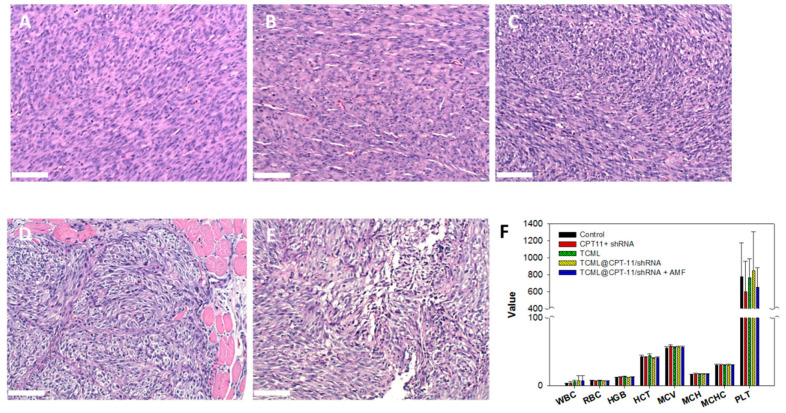
The hematoxylin-eosin (H&E) staining of tumor tissues explanted from nude mice after treatment with normal saline (control) (**A**), CPT-11 + shRNA (**B**), TCML@CPT-11 (**C**), TCML@CPT-11/shRNA (**D**), and TCML@CPT11/shRNA + AMF (**E**), and the hematological parameters of blood samples withdrawn from the mice (**F**) on day 22 after tumor implantation. Bar = 100 μm.

The hematoxylin-eosin (H&E) staining of harvested tumor tissue on day 22 revealed substantial necrosis of the cancer cells for TCML@CPT11-shRNA + AMF treatment when compared with other groups (Figure 12A–E). Furthermore, the application of TCML-CPT11-shRNA + AMF did not significantly alter the hematologic parameters when compared with the control group, based on blood samples withdrawn from the mice on day 22 (Figure 12F), which confirms the safety of the treatment. The expression levels of SLP2 in vivo were determined by immunohistochemical staining of the tissue slices from each group and presented in Figure S2 (Supplementary Materials), which supports effective SLP2 gene silencing in vivo.

## 4. Conclusions

In this study, TCML liposomal system was developed for the co-delivery of CPT-11 and SLP2 shRNA, and successfully used in glioma treatment after AMF induction. In addition to the chemotherapeutic drug CPT-11, CMNPs can be co-encapsulated in the liposomes to achieve magnetic targeting and triggered drug release, which can enhance the chemotherapeutic efficacy of AMF-responsive drug delivery. The cationic nature of TCMLs can facilitate complexation with SLP2 shRNA plasmids and the TCML/shRNA can provide very high transfection efficiency for SLP2 gene silencing to inhibit U87 migration and reduce the invasiveness of glioblastoma cells. In the animal experiment, the TCML@CPT-11/shRNA + AMF treatment demonstrated excellent antitumor efficacy in nude mice xenografts, showing significantly prolonged survival times and a substantially repressed tumor growth rate. This treatment is also safe, judging from the insignificant change in body weight and hematologic parameters. This functional liposomal system for drug/gene delivery will be suitable for glioblastoma treatment to provide enhanced therapeutic efficacy with minimal adverse effects.

## Figures and Tables

**Table 1:** The survival times for U87 tumor-bearing mice after different treatments (*n* = 5).

Group	Median (Days)	Average ^1^
Control	20	25.0 ± 8.2
CPT-11 + shRNA	29	29.5 ± 5.4 ^α^
TCML	22	25.2 ± 4.1
TCML@CPT-11/shRNA	29	29.6 ± 2.5 ^α^
TCML@CPT-11/shRNA + AMF	36	37.2 ± 4.1 ^α,β,γ,δ^

^1^ Mean ± standard deviation (SD). ^α^ *p* < 0.05 compared with control, ^β^ *p* < 0.05 compared with CPT-11 + shRNA, ^γ^ *p* < 0.05 compared with TCML, ^δ^ *p* < 0.05 compared with TCML@CPT-11/shRNA + AMF.

## Data Availability

The data presented in this study are available on request from the corresponding author.

## Supplementary Materials

The following supporting information can be download at https://www.mdpi.com/article/10.3390/pharmaceutics15041169/s1: Figure S1: The SLP2 gene silencing in vitro; Figure S2: The SLP2 gene silencing in vivo.
