# Supplementary material for: Thermosensitive Cationic Magnetic Liposomes for Thermoresponsive Delivery of CPT-11 and SLP2 shRNA in Glioblastoma Treatment
Source: Pharmaceutics. 2023 Apr 6;15(4):1169. doi: 10.3390/pharmaceutics15041169 (PMC10145679; doi:10.3390/pharmaceutics15041169)
Supplement: Supplementary file 1 [file pharmaceutics-15-01169-s001.zip › pharmaceutics-2276786-supplementary.pdf]

Supplementary Materials

Thermosensitive Cationic Magnetic Liposomes for Thermo-Responsive Delivery of CPT-11 and SLP2 shRNA in Glioblastoma Treatment

SLP2 gene silencing *in vitro*

The Western immunoblot analysis was used to study SLP2 gene silencing i*n vitro*. 2 x 10^6 U87 cells were cultured in a T75 flask for 24 h and treated with medium, TCML, shRNA or TCML@shRNA for 3 days. The harvested U87 cells were lysis to extract total protein and the protein concentration in supernatant was determined. After heat denaturation, the protein was separated on a sodium dodecyl sulfate polyacrylamide gel by electrophoresis. The gel was transferred to a polyvinylidene fluoride membrane, blotted with primary antibody and probed with horseradish peroxidase-conjugated secondary antibody and incubated with a substrate for color development. The formed complexes were detected using a gel imaging system and analyzed of protein band intensity. As shown from Figure S1, the relative protein expression for TCML/shRNA is significantly reduced when compared with other groups, confirming the silencing of SLP2 gene *in vitro*.

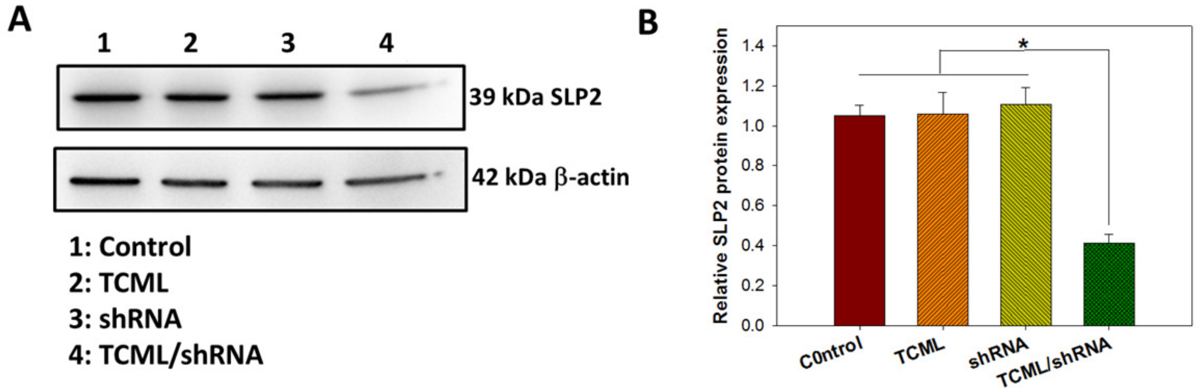

Figure S1. (A) The SLP2 protein synthesis from the Western blot analysis after U87 cells were treated with medium (control), TCML, shRNA and TCML/shRNA for 72 h, and (B) the relative SLP2 protein expression using β-actin as a loading control. * $p < 0.05$.

SLP2 gene silencing *in vivo*

For SLP2 gene silencing *in vivo*, the SLP2 protein expression in the tumor section was determined by immunohistochemical staining. After incubating the sample with rabbit primary antibody against SLP2 for 24 h, a horseradish peroxidase-conjugated anti-rabbit secondary antibody was used for color development. The slice was counterstained with hematoxylin for nuclei and examined under an inverted microscope. The SLP2 protein expression level was quantified from the area percentage of the immune-reactive area within the region of interest in each picture frame. As shown form Figure S2, there is significant difference in SLP2 protein

expression when TCML@CPT-11/shRNA was used for treatment, endorsing the *in vivo* silencing of SLP 2 gene.

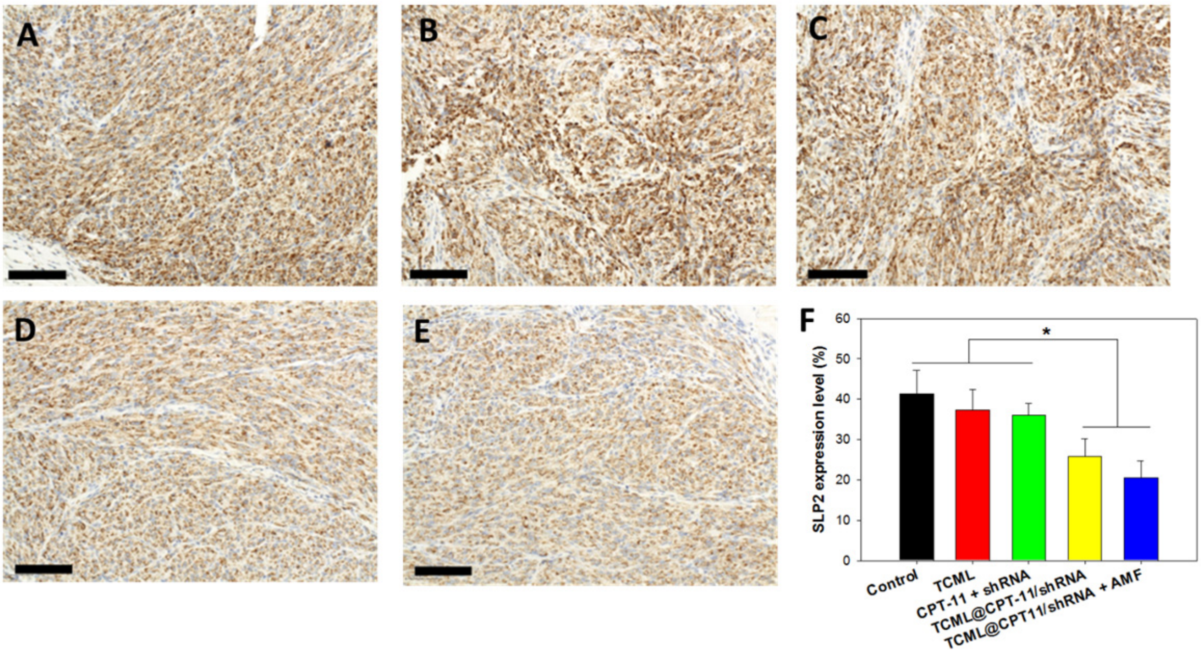

Figure S2. The immunohistochemical staining of SLP2 in tumor sections after explanting tumor sample from nude mice that is treated with normal saline (control) (A), CPT-11 + shRNA (B), TCML@CPT-11 (C), TCML@CPT-11/shRNA (D) or TCML@CPT11/shRNA + AMF (E). (F) The SLP2 expression level calculated form the area percentage of immune-reactive area within the region of interest in each picture frame. Bar = 50 μm. * $p < 0.05$.
